# Association between Osteoporosis and Previous Statin Use: A Nested Case-Control Study

**DOI:** 10.3390/ijerph182211902

**Published:** 2021-11-12

**Authors:** So Young Kim, Dae Myoung Yoo, Chanyang Min, Ji Hee Kim, Mi Jung Kwon, Joo-Hee Kim, Hyo Geun Choi

**Affiliations:** 1 Department of Otorhinolaryngology-Head & Neck Surgery, CHA Bundang Medical Center, CHA University, Seongnam 13496, Korea; sossi81@hanmail.net; 2 Hallym Data Science Laboratory, Hallym University College of Medicine, Anyang 14068, Korea; ydm1285@naver.com (D.M.Y.); joicemin@naver.com (C.M.); 3 Graduate School of Public Health, Seoul National University, Seoul 08826, Korea; 4 Department of Neurosurgery, Hallym University College of Medicine, Anyang 14068, Korea; kimjihee.ns@gmail.com; 5 Department of Pathology, Hallym University College of Medicine, Anyang 14068, Korea; mulank@hanmail.net; 6 Division of Pulmonary, Allergy, and Critical Care Medicine, Department of Medicine, Hallym University College of Medicine, Anyang 14068, Korea; luxjhee@gmail.com; 7 Department of Otorhinolaryngology-Head & Neck Surgery, Hallym University College of Medicine, Anyang 14068, Korea

**Keywords:** osteoporosis, hydroxymethylglutaryl-CoA reductase inhibitors, risk factors, cohort studies

## Abstract

The relationship between statin use and osteoporosis is controversial; therefore, this study aimed to investigate this association. The ≥40-year-old population of the Korean National Health Insurance Service Health Screening Cohort was enrolled. The 68,592 osteoporosis patients were matched 1:1 with control participants for age, sex, income, and region of residence using propensity score matching. The histories of statin use for two years before the diagnosis of osteoporosis (index date) in the osteoporosis and control groups were compared using conditional/unconditional logistic regression. An increased number of days of statin use was not associated with osteoporosis (adjusted OR (aOR) = 0.97, 95% confidence interval (95% CI) = 0.94–1.00, *p* = 0.052). In the subgroup analyses, a large number of days of statin use was related to a reduced rate of osteoporosis in the <60-year-old female group, while the opposite was true in the ≥60-year-old female group. Both lipophilic and hydrophilic statins were related to a decreased rate of osteoporosis in the <60-year-old female group. Lipophilic statins, but not hydrophilic statins, were associated with an increased rate of osteoporosis in the ≥60-year-old female group. Statin use showed different associations in middle-aged and elderly women.

## 1. Introduction

Osteoporosis is defined as a skeletal disorder with decreased bone strength that increases the risk of fracture [1]. With an aging population, the prevalence of osteoporosis has risen to as high as 46.9% (95% confidence interval (95% CI) = 45.4–48.4) in women upon diagnosis based on a comprehensive evaluation of fracture risk [2]. Multiple pathophysiological factors can induce osteoporotic changes by compromising the intrinsic bone repair mechanism or exaggerating the bone remodeling rates [3]. A few plausible mechanisms that cause systemic immune imbalance, and inflammatory responses have been acknowledged as factors associated with osteoporosis, such as interactions between bone and the immune system and cellular senescence [4]. In addition, there are sex-specific differences in the pathophysiology of osteoporosis due to the distinct bone physiology and sex hormone responses [5].

Statins are lipid-lowering agents that inhibit hydroxymethylglutaryl-CoA reductase, which is a rate-limiting enzyme in cholesterol synthesis pathways [6]. In addition to their effects on lipid profiles, statins have pleiotropic effects via anti-inflammatory, antioxidative, and immunomodulatory activities [7]. Through these versatile mechanisms of action, the clinical indications for statins have been widened from dyslipidemia to coronary artery disease and stroke [8]. The comorbidities of dyslipidemia and cardiovascular diseases are factors associated with osteoporosis [9,10]. In addition, inflammation and immune dysfunctions are also accompanied by osteoporosis [11,12]. Therefore, it can be presumed that statins have beneficial effects on osteoporosis.

In line with this, several previous studies have suggested an association between statins and an increased risk of fracture and decreased bone mineral density (BMD) [13,14]. Moreover, a few previous studies have shown a decreased risk of osteoporosis in relation to previous statin use [15]. However, other studies have reported an increased risk of osteoporosis or no association between osteoporosis and statin use [14,16]. The differences in study populations and types and durations of statins could result in discrepancies in the effects of statins on osteoporosis.

We supposed that the effects of statins on osteoporosis could be different according to both patient factors and factors related to statin medications. A recent study reported a beneficial effect of statins on osteoporosis at low doses but a hazardous effect of statins on osteoporosis at high doses [17]. Thus, we summed the total use dates for two years before the index date of this study. Another study reported sex differences in the effects of statins [18]. Therefore, we analyzed subgroups according to age and sex.

## 2. Materials and Methods

### 2.1. Ethics

The ethics committee of Hallym University (23 October 2019) approved this study. Written informed consent was waived by the Institutional Review Board. All analyses adhered to the guidelines and regulations of the ethics committee of Hallym University.

### 2.2. Study Population and Participant Selection

**Figure 1:**
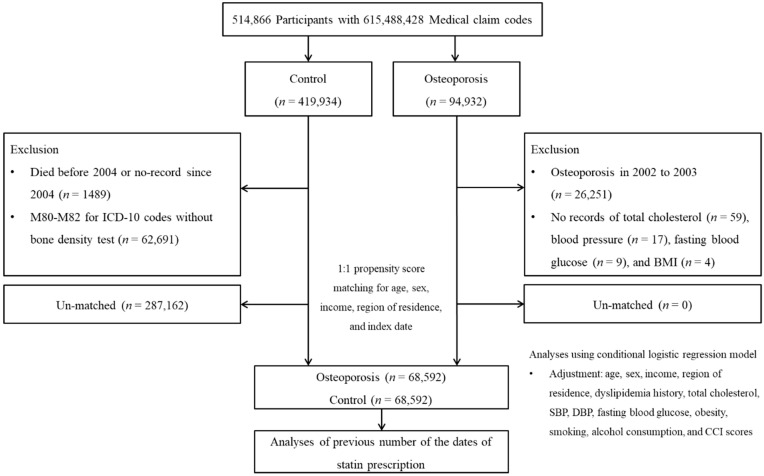
A schematic illustration of the participant selection process that was used in the present study. Of a total of 514,866 participants, 68,592 osteoporosis participants were matched 1:1 with 68,592 control participants for age, sex, income, and region of residence using propensity score matching. Abbreviation: BMI, body mass index.

A detailed description of the Korean National Health Insurance Service Health Screening Cohort data is described elsewhere [19,20]. Osteoporosis participants were selected from among 514,866 participants with 615,488,428 medical claim codes (*n* = 94,932). The control group included all participants without osteoporosis (*n* = 419,934). To assess statin use dates for two years, we excluded participants with osteoporosis who were diagnosed with osteoporosis between 2002 and 2003 (*n* = 26,251). Among the control participants, we excluded those who died before 2004 or had no records after 2004 (*n* = 1489) and those treated with the ICD-10 codes M80-M82 without a bone density test (*n* = 62,691). Osteoporosis participants were excluded if they had no records of total cholesterol level (*n* = 59), blood pressure (*n* = 17), fasting blood glucose level (*n* = 9), or body mass index (BMI, kg/m^2^; *n* = 4). Osteoporosis participants were matched 1:1 with control participants for age, sex, income, and region of residence using propensity score matching. The index date of each osteoporosis participant was set as the time of treatment for osteoporosis. The index date of the control participants was set as the index date of their matched osteoporosis participants. Therefore, each matched osteoporosis participant and control participant had the same index date. During the matching process, 287,162 control participants were excluded. Finally, 68,592 osteoporosis participants were matched 1:1 with 68,592 control participants (Figure 1).

### 2.3. Exposure (Dates of Statin Use)

The sum of the total dates of statin use was counted as continuous for two years (730 days) before the index dates. The statins investigated for this study included atorvastatin, fluvastatin, lovastatin, pitavastatin, pravastatin, rosuvastatin, and simvastatin. Pravastatin and rosuvastatin were categorized as hydrophilic statins, and atorvastatin, fluvastatin, lovastatin, pitavastatin, and simvastatin were categorized as lipophilic statins.

### 2.4. Outcome (Osteoporosis)

Osteoporosis was defined if the participants were diagnosed with M80 (osteoporosis with pathological fracture), M81 (osteoporosis without pathological fracture), or M82 (osteoporosis in diseases classified elsewhere) ≥2 times using ICD-10 codes for bone density tests performed using X-ray or computed tomography (CT) (claim codes: E7001–E7004 and HC341–HC345).

### 2.5. Covariates

Age groups were divided into five-year intervals: 40–44, 45–49, 50–54, …, and 85+ years old (total of 10 age groups). Income groups were classified into five classes (class 1 (lowest income)–5 (highest income)). The region of residence was grouped into urban and rural areas according to our previous study [21]. Tobacco smoking status, alcohol consumption, and obesity using BMI (kg/m^2^) were categorized in the same way as they were in our previous study [22]. Systolic/diastolic blood pressure, fasting blood glucose level, and total cholesterol level were measured. Dyslipidemia was defined as the presence of ICD-10 code E78 ≥ 2 times before the index date.

The Charlson comorbidity index (CCI) is used widely to measure disease burden using 17 comorbidities as the continuous variable (0 (no comorbidities) through 29 (multiple comorbidities)) [23]. Among them, we excluded thyroid cancer.

### 2.6. Statistical Analyses

The general characteristics of the osteoporosis and control groups were compared using the Wilcoxon rank-sum test.

To analyze the odds ratios (ORs) with 95% CIs of the dates of statin use (one year) for osteoporosis, unconditional logistic regression was used. In this analysis, model 1 (age, sex, income, and region of residence), model 2 (adjusted for model 1 plus dyslipidemia history, total cholesterol level, SBP, DBP, and fasting blood glucose level), and model 3 (adjusted for model 2 plus obesity, smoking status, alcohol consumption, and CCI scores) were calculated. Additionally, we performed analyses according to the type of statin (hydrophilic or lipophilic).

For the subgroup analyses, we divided participants by age, sex, income, and region of residence (<60 years old and ≥60 years old; male and female; low income [1,2,3] and high income [4,5]; urban and rural) and analyzed models 1, 2, and 3. We additionally performed subgroup analyses according to obesity, smoking status, alcohol consumption, total cholesterol level, blood pressure, and fasting blood glucose level using unconditional logistic regression.

Two-tailed analyses were performed, and significance was defined as *p*-values less than 0.05. SAS version 9.4 (SAS Institute Inc., Cary, NC, USA) was used for the statistical analyses.

## 3. Results

The mean number of days of statin use was 56.9 (standard deviation (SD) = 162.8) and 50.6 (SD = 154.1) days for the osteoporosis and control groups, respectively (*p* < 0.001, Table 1). The prevalence of dyslipidemia was higher in the osteoporosis group than in the control group (26.5% (18, 183/68, 592) vs. 22.9% (15, 716/68, 592), *p* < 0.001). The levels of total cholesterol, SBP, DBP, and fasting blood glucose were different in the osteoporosis and control groups (all *p* < 0.001). The distributions of BMI groups, smoking status, alcohol consumption, and CCI score were different in the osteoporosis and control groups (all *p* < 0.001).

The rate of osteoporosis was 1.04 times higher according to an increased number of days of statin use in model 1 (95% CI = 1.02–1.07, *p* = 0.001, Table 2). However, the rate of osteoporosis was 0.96 times higher in the patients with more days of statin use in model 2 (95% CI = 0.93–0.98, *p* = 0.002). When adjusting for lifestyle factors and past medical histories in model 3, osteoporosis was not associated with the number of days of statin use in the total study population (adjusted OR (aOR) = 0.97, 95% CI = 0.94–1.00, *p* = 0.052).

In the age and sex subgroups, the <60-year-old female group showed a reduced rate of osteoporosis associated with the number of days of statin use (aOR = 0.81, 95% CI = 0.77–0.85, *p* < 0.001). On the other hand, the ≥60-year-old female group demonstrated an increased rate of osteoporosis associated with the number of days of statin use (aOR = 1.37, 95% CI = 1.30–1.46, *p* < 0.001). Among the income or region of residence subgroups, the urban group showed a decreased rate of osteoporosis related to the number of days of statin use (aOR = 0.93, 95% CI = 0.89–0.97, *p* = 0.001). Age groups were subdivided into 10-year intervals to analyze the relationship between osteoporosis and statin use according to age subgroups (Table S1). The 50- to 59-year-old female group and the 60- to 69-year-old female group showed a decreased rate of osteoporosis related to the number of days of statin use (aOR = 0.89, 95% CI = 0.83–0.96, *p* = 0.002 for the 50- to 59-year-old female group and aOR = 0.96, 95% CI = 0.90–0.99, *p* = 0.017 for the 60- to 69-year-old female group). In contrast, the 70- to 79-year-old female group and the ≥80-year-old female group showed an increased rate of osteoporosis associated with the number of days of statin use (aOR = 1.43, 95% CI = 1.31–1.56, *p* < 0.001 for the 70- to 79-year-old female group and aOR = 1.27, 95% CI = 1.01–1.60, *p* = 0.044 for the ≥ 80-year-old female group).

Moreover, subgroups with obesity, alcohol consumption ≥1 time a week, total cholesterol <200 mg/dL, both normal and high blood pressure, and fasting blood glucose <100 mg/dL showed a reduced rate of osteoporosis related to the number of days of statin use (Table 3).

The type of statins were specified as hydrophilic or lipophilic, and their associations with osteoporosis were further analyzed (Tables S2 and S3). Hydrophilic statins were associated with a decreased rate of osteoporosis in the <60-year-old female group (aOR = 0.86, 95% CI = 0.78–0.96, *p* = 0.005). Lipophilic statins were also related to a decreased rate of osteoporosis in the <60-year-old female group (aOR = 0.81, 95% CI = 0.77–0.85, *p* < 0.001). However, the ≥60-year-old female group showed an increased rate of osteoporosis related to the number of days of lipophilic statin use (aOR = 1.41, 95% CI = 1.33–1.50, *p* < 0.001).

## 4. Discussion

Previous statin use was not related to osteoporosis overall. However, prior statin use was associated with a decreased rate of osteoporosis in middle-aged women and in some subgroups, including the urban residence, obese, and alcohol consumption groups. On the contrary, older women showed an increased rate of osteoporosis associated with prior statin use. The decreased rate of osteoporosis related to prior statin use in middle-aged women was valid for both hydrophilic and lipophilic statin use. However, the increased rate of osteoporosis related to prior statin use in older women was consistent only with lipophilic statin use. The present results improved the previous findings on the association of statins with osteoporosis by demonstrating the different relations according to age, sex, and type of statin.

Several clinical studies have suggested that both types of statins have protective effects on osteoporosis, though with some conflicting results [15,17,24]. A meta-analysis demonstrated that statin use was related to increased BMD [15]. A nationwide retrospective population-based cohort study in Taiwan described a 48% decreased risk of new-onset osteoporosis in statin users compared to nonstatin users (adjusted hazard ratio = 0.52, 95% CI = 0.50–0.54) [24]. Another population-based cohort study in Taiwan demonstrated no association of new-onset osteoporotic fracture with statin use [25]. On the contrary, a cross-sectional retrospective study in Austria reported an increased rate of osteoporosis related to statin use in the overall study population (aOR = 3.62, 95% CI = 3.55–3.69) [17]. However, their study population was old, with a mean age of 65.2 years old for men and 69.02 years old for women, and low-dose statin use (0–10 mg/day) was associated with a decreased rate of osteoporosis [17]. In summary, statins might be beneficial in protecting against osteoporosis, but they might have some harmful effects on osteoporosis under some circumstances, such as at high doses or in specific age or sex groups.

Statins may protect against osteoporosis by promoting osteogenesis and preventing osteoporotic changes. Statins accelerate the differentiation of mesenchymal cells to osteoblasts by upregulating BMP-2 and have antiapoptotic effects on osteoblasts [26]. In addition, statins inhibit osteoclast activation and differentiation [26]. A few preclinical studies have reported the anabolic effects of statins on bone [26,27]. For instance, in an apolipoprotein E-deficient (apoE^−/−^) mouse study, atorvastatin administration for as long as 12 weeks increased bone mass and improved bone microarchitecture in trabecular bone and increased the mRNA expression of the serum bone formation marker osteocalcin [27]. Therefore, in addition to the lipid-lowering effects, the osteogenic effects of statins may lower the risk of osteoporosis.

On the contrary, statins could increase the risk of osteoporosis by disturbing the synthesis of estrogen, especially in elderly women. Because cholesterol is a precursor for sex hormones, inhibiting cholesterol synthesis with statins could also decrease the levels of sex hormones. Indeed, the intragastric administration of simvastatin decreased the serum levels of estradiol, progesterone, and testosterone, as well as those of total cholesterol, low-density lipoprotein cholesterol, and triglycerides in rats [28,29]. Estrogen deficiency has been acknowledged as one of the major causes of osteoporosis in postmenopausal women [30]. It has been suggested that increased bone resorption due to estrogen deprivation is a main pathophysiology of osteoporosis in postmenopausal women, while a decrease in osteoblast activity is a main pathophysiology of osteoporosis in men [15]. Thus, the estrogen-lowering effect of statins could elevate the risk of osteoporosis in postmenopausal or elderly women. In the present study, the elderly woman group showed association of prior statin use with an increased rate of osteoporosis. In addition to the estrogen-lowering effect of statins, survival bias could influence this positive relation between prior statin use and osteoporosis. However, the average survival age was 64.3 years old (standard deviation (SD) = 9.1) for the osteoporosis group and 61.7 years old (SD = 8.5) for the control group.

Only lipophilic statins demonstrated a positive association between statin use and osteoporosis in elderly women in the present study. The high absorption rate of lipophilic statins could increase their bioavailability and distribution in the body compared to hydrophilic statins, which could elevate the effects on estrogen deprivation and osteoporosis in elderly women. Hydrophilic statins have been reported to have a decreased absorption rate and dependency on the cytochrome P450 enzyme and showed fewer adverse effects than lipophilic statins [31]. In addition to osteoporosis, a previous study reported an increased rate of coronary artery disease associated with lipophilic statins but not hydrophilic statins [32]. The different tissue selectivities and bioavailabilities could result in different associations of statins with osteoporosis, especially in vulnerable populations, such as elderly women.

We used a large population database encompassing various socioeconomic factors, comorbidities, lifestyle factors, and laboratory data. The matched control group was randomly selected, and various potential confounders were adjusted. Thus, potential bias from the selection process or confounders could be attenuated. However, due to limited database information, the BMD and levels of bone metabolic factors could not be measured. Because osteoporosis was defined using diagnostic codes (M80–M82), the severity of osteoporosis might be heterogeneous in the osteoporosis group. In addition, the participants who did not visit the clinic and asymptomatic osteoporotic patients could have been misclassified as the control group in this study. For statin use, we could not check for compliance with statin prescriptions. Although many confounders were adjusted for in this study, there could be unconfirmed confounding factors such as other medication histories. Last, due to the retrospective study design, the causality between prior statin use and osteoporosis is elusive. A prospective study on the effect of statin use on osteoporosis is warranted.

## 5. Conclusions

Previous statin use was not related to osteoporosis in the adult population. However, prior statin use was associated with a decreased risk of osteoporosis in middle-aged women. In contrast, elderly women showed an increased risk of osteoporosis related to previous statin use, which was solid in lipophilic statin use.

## Figures and Tables

**Table 1:** General characteristics of the participants.

Characteristics		Participants			
		Total	Osteoporosis	Control	*p*-Value
Age (years old, *n*, %)					<0.001 *
	40–44	778 (0.6)	389 (0.6)	389 (0.6)	
	45–49	6540 (4.8)	3270 (4.8)	3270 (4.8)	
	50–54	16,350 (11.9)	8175 (11.9)	8175 (11.9)	
	55–59	23,542 (17.2)	9582 (40.7)	13,960 (59.3)	
	60–64	31,731 (23.1)	11,034 (34.8)	20,697 (65.2)	
	65–69	24,109 (17.6)	14,995 (21.9)	9114 (13.3)	
	70–74	18,960 (13.8)	11,968 (17.5)	6992 (10.2)	
	75–79	10,661 (7.8)	6505 (9.5)	4156 (6.1)	
	80–84	3875 (2.8)	2293 (3.3)	1582 (2.3)	
	85+	638 (0.5)	381 (0.6)	257 (0.4)	
Sex (*n*, %)					1.000
	Male	17,694 (12.9)	8847 (12.9)	8847 (12.9)	
	Female	119,490 (87.1)	59,745 (87.1)	59,745 (87.1)	
Income (*n*, %)					<0.001 *
	1 (lowest)	25,221 (18.4)	13,218 (19.3)	12,003 (17.5)	
	2	19,679 (14.3)	9751 (14.2)	9928 (14.5)	
	3	21,440 (15.6)	10,641 (15.5)	10,799 (15.7)	
	4	28,817 (21.0)	13,681 (20.0)	15,136 (22.1)	
	5 (highest)	42,027 (30.6)	21,301 (31.1)	20,726 (30.2)	
Region of residence (*n*, %)					<0.001 *
	Urban	54,018 (39.4)	26,448 (38.6)	27,570 (40.2)	
	Rural	83,166 (60.6)	42,144 (61.4)	41,022 (59.8)	
Total cholesterol (mg/dL, mean, SD)		204.7 (39.0)	203.7 (38.7)	205.7 (39.3)	<0.001 *
SBP (mmHg, mean, SD)		127.6 (18.0)	127.3 (17.8)	127.9 (18.3)	<0.001 *
DBP (mmHg, mean, SD)		78.1 (11.1)	77.8 (10.9)	78.5 (11.2)	<0.001 *
Fasting blood glucose (mg/dL, mean, SD)		99.4 (30.0)	98.1 (27.6)	100.7 (32.1)	<0.001 *
Obesity (*n*, %) ‡					<0.001 *
	Underweight	3806 (2.8)	2281 (3.3)	1525 (2.2)	
	Normal	48,800 (35.6)	25,762 (37.6)	23,038 (33.6)	
	Overweight	36,049 (26.3)	17,864 (26.0)	18,185 (26.5)	
	Obese I	43,449 (31.7)	20,602 (30.0)	22,847 (33.3)	
	Obese II	5080 (3.7)	2083 (3.0)	2997 (4.4)	
Smoking status (*n*, %)					<0.001 *
	Nonsmoker	125,177 (91.3)	62,797 (91.6)	62,380 (90.9)	
	Past smoker	4765 (3.5)	2363 (3.5)	2402 (3.5)	
	Current smoker	7242 (5.3)	3432 (5.0)	3810 (5.6)	
Alcohol consumption (*n*, %)					<0.001 *
	<1 time a week	119,132 (86.8)	60,336 (88.0)	58,796 (85.7)	
	≥1 time a week	18,052 (13.2)	8256 (12.0)	9796 (14.3)	
CCI score (score, *n*, %)					<0.001 *
	0	89,612 (65.3)	42,465 (61.9)	47,147 (68.7)	
	1	21,208 (15.5)	11,807 (17.2)	9401 (13.7)	
	2	11,954 (8.7)	6622 (9.7)	5332 (7.8)	
	3	6149 (4.5)	3425 (5.0)	2724 (4.0)	
	≥4	8261 (6.0)	4273 (6.2)	3988 (5.8)	
Dyslipidemia (*n*, %)		33,899 (24.7)	18,183 (26.5)	15,716 (22.9)	<0.001 *
Dates of statin use (days, mean, SD)		53.7 (158.5)	56.9 (162.8)	50.6 (154.1)	<0.001 *

Abbreviations: CCI, Charlson comorbidity index; DBP, diastolic blood pressure; SBP, systolic blood pressure; SD, standard deviation. * Wilcoxon rank-sum test. Significance at *p* < 0.05. ‡ Obesity (BMI, body mass index, kg/m^2^) was categorized as <18.5 (underweight), ≥18.5 to <23 (normal), ≥23 to < 25 (overweight), ≥25 to <30 (obese I), and ≥30 (obese II).

**Table 2:** Odds ratios (95% confidence interval) of the date of statin use (one year) for osteoporosis with subgroup analyses according to age, sex, income, and region of residence.

Characteristics		Odds Ratios for Osteoporosis					
		Model 1 †	*p*-Value	Model 2 ‡	*p*-Value	Model 3 §	*p*-Value
Total participants (*n* = 137,184)							
	Statin prescription (one year)	1.04 (1.02–1.07)	0.001 *	0.96 (0.93–0.98)	0.002 *	0.97 (0.94–1.00)	0.052
Age < 60 years old, males (*n* = 4,178)							
	Statin prescription (one year)	1.11 (0.96–1.29)	0.172	0.97 (0.82–1.15)	0.727	0.99 (0.84–1.18)	0.942
Age < 60 years old, females (*n* = 74,763)							
	Statin prescription (one year)	0.85 (0.82–0.88)	<0.001 *	0.80 (0.76–0.83)	<0.001 *	0.81 (0.77–0.85)	<0.001 *
Age ≥ 60 years old, males (*n* = 13,516)							
	Statin prescription (one year)	0.99 (0.93–1.05)	0.705	0.95 (0.88–1.02)	0.178	0.96 (0.89–1.03)	0.281
Age ≥ 60 years old, females (*n* = 44,727)							
	Statin prescription (one year)	1.76 (1.67–1.86)	<0.001 *	1.36 (1.28–1.44)	<0.001 *	1.37 (1.30–1.46)	<0.001 *
Low income (*n* = 66,340)							
	Statin use (one year)	1.05 (1.01–1.09)	0.010 *	0.95 (0.91–0.99)	0.022 *	0.97 (0.93–1.01)	0.170
High income (*n* = 70,844)							
	Statin use (one year)	1.04 (1.00–1.07)	0.043 *	0.96 (0.92–0.99)	0.025	0.97 (0.94–1.01)	0.147
Urban (*n* = 54,018)							
	Statin use (one year)	1.02 (0.98–1.06)	0.302	0.92 (0.88–0.96)	<0.001 *	0.93 (0.89–0.97)	0.001 *
Rural (*n* = 83,166)							
	Statin use (one year)	1.08 (1.04–1.11)	<0.001 *	0.99 (0.96–1.03)	0.703	1.01 (0.97–1.05)	0.625

Abbreviations: CCI, Charlson comorbidity index; DBP, diastolic blood pressure; SBP, systolic blood pressure. * Logistic regression, significance at *p* < 0.05. † Model 1 was adjusted for age, sex, income, and region of residence. ‡ Model 2 was adjusted for model 1 plus dyslipidemia history, total cholesterol, SBP, DBP, and fasting blood glucose. § Model 3 was adjusted for model 2 plus obesity, smoking, alcohol consumption, and CCI scores.

**Table 3:** Odds ratios (95% confidence interval) of the date of statin use (one year) for osteoporosis in each subgroup according to obesity, smoking, alcohol consumption, total cholesterol, blood pressure, and fasting blood glucose.

Characteristics		Odds Ratios of Statins Use (One Year) for Osteoporosis					
		Model 1 †	*p*-Value	Model 2 ‡	*p*-Value	Model 3 §	*p*-Value
Obesity							
	Underweight (*n* = 3806)	1.44 (1.12–1.84)	0.004 *	1.14 (0.86–1.52)	0.369	1.14 (0.86–1.51)	0.374
	Normal weight (*n* = 48,800)	1.08 (1.03–1.14)	0.002 *	0.97 (0.91–1.02)	0.217	0.96 (0.91–1.02)	0.179
	Overweight (*n* = 36,049)	1.10 (1.05–1.15)	<0.001 *	0.99 (0.94–1.05)	0.817	0.99 (0.94–1.05)	0.796
	Obese (*n* = 48,529)	1.04 (1.01–1.08)	0.019 *	0.95 (0.91–0.99)	0.021 *	0.95 (0.91–0.99)	0.025 *
Smoking							
	Nonsmoker (*n* = 125,177)	1.04 (1.01–1.07)	0.003 *	0.96 (0.93–0.98)	0.003 *	0.97 (0.94–1.00)	0.066
	Past or current smoker (*n* = 12,007)	1.05 (0.97–1.13)	0.232	0.95 (0.87–1.04)	0.245	0.97 (0.89–1.06)	0.527
Alcohol consumption							
	<1 time a week (*n* = 119,132)	1.06 (1.03–1.09)	<0.001 *	0.97 (0.94–1.00)	0.032 *	0.98 (0.95–1.01)	0.269
	≥1 time a week (*n* = 18,052)	0.99 (0.93–1.05)	0.707	0.92 (0.85–0.98)	0.015 *	0.93 (0.87–1.00)	0.045 *
Total cholesterol (mg/dL)							
	<200 (*n* = 64,727)	1.01 (0.98–1.04)	0.572	0.92 (0.88–0.95)	<0.001 *	0.94 (0.90–0.97)	0.001 *
	≥200 to <240 (*n* = 48,835)	1.08 (1.03–1.14)	0.002 *	1.00 (0.94–1.05)	0.877	1.01 (0.95–1.07)	0.819
	≥240 (*n* = 23,622)	1.13 (1.07–1.21)	<0.001 *	1.04 (0.97–1.12)	0.225	1.06 (0.99–1.13)	0.119
Blood pressure (mmHg)							
	SBP < 140 and DBP < 90 (*n* = 97,751)	0.99 (0.97–1.02)	0.674	0.91 (0.88–0.94)	<0.001 *	0.93 (0.90–0.96)	<0.001 *
	SBP ≥ 140 or DBP ≥ 90 (*n* = 39,433)	1.17 (1.12–1.22)	<0.001 *	1.05 (1.00–1.11)	0.046 *	1.07 (1.01–1.12)	0.013 *
Fasting blood glucose (mg/dL)							
	<100 (*n* = 90,834)	1.07 (1.03–1.11)	<0.001 *	0.94 (0.90–0.98)	0.002 *	0.96 (0.92–0.99)	0.024 *
	≥100 (*n* = 46,350)	1.07 (1.03–1.11)	<0.001 *	0.98 (0.94–1.02)	0.386	1.00 (0.96–1.04)	0.845

Abbreviations: CCI, Charlson comorbidity index; DBP, diastolic blood pressure; SBP, systolic blood pressure. * Logistic regression, significance at *p* < 0.05. † Model 1 was adjusted for age, sex, income, and region of residence. ‡ Model 2 was adjusted for model 1 plus dyslipidemia history, total cholesterol, SBP, DBP, and fasting blood glucose. § Model 3 was adjusted for model 2 plus, obesity, smoking, alcohol consumption, and CCI scores.

## Data Availability

Releasing data from this research is not legally permitted. All data are available from the database of the Korea Center for Disease Control and Prevention. The Korea Center for Disease Control and Prevention allows data access, at a particular cost, for any researcher who promises to follow the research ethics. The data of this article can be downloaded from the website after agreeing to follow the research ethics.

## Supplementary Materials

The following are available online at https://www.mdpi.com/article/10.3390/ijerph182211902/s1, Table S1. Subgroup analyses of the odds ratios (95% confidence interval) of the date of statin use (one year) for osteoporosis according to specific age groups and sex; Table S2. Odds ratios (95% confidence interval) of the date of hydrophilic statin use (one year) for osteoporosis with subgroup analyses according to age, sex, income, and region of residence; Table S3. Odds ratios (95% confidence interval) of the date of lipophilic statin use (one year) for osteoporosis with subgroup analyses according to age, sex, income, and region of residence.
