# Supplementary material for: Association between Osteoporosis and Previous Statin Use: A Nested Case-Control Study
Source: Int J Environ Res Public Health. 2021 Nov 12;18(22):11902. doi: 10.3390/ijerph182211902 (PMC8620647; doi:10.3390/ijerph182211902)
Supplement: Supplementary file 1 [file ijerph-18-11902-s001.zip › ijerph-1430715-supplementary.pdf]

Table S1. Subgroup analyses of odds ratios (95% confidence interval) of the date of statin prescription (1 year) for osteoporosis according to specific age group and sex

Characteristics	Odds ratios for osteoporosis					
	Model 1†	P-value	Model 2‡	P-value	Model 3§	P-value
Age 40-49 years old, males (n = 348)						
Statin prescription (1 year)	1.22 (0.54-2.73)	0.633	0.98 (0.38-2.51)	0.966	0.90 (0.34-2.36)	0.832
Age 40-49 years old, females (n = 6,970)						
Statin prescription (1 year)	1.69 (1.14-2.50)	0.009*	1.15 (0.76-1.74)	0.522	1.15 (0.75-1.77)	0.511
Age 50-59 years old, males (n = 2,074)						
Statin prescription (1 year)	1.23 (0.97-1.56)	0.095	1.12 (0.86-1.45)	0.400	1.16 (0.89-1.52)	0.267
Age 50-59 years old, females (n = 37,818)						
Statin prescription (1 year)	0.95 (0.89-1.01)	0.101	0.88 (0.82-0.94)	<0.001*	0.89 (0.83-0.96)	0.002*
Age 60-69 years old, males (n = 4,812)						
Statin prescription (1 year)	1.01 (0.90-1.14)	0.873	0.91 (0.80-1.04)	0.181	0.94 (0.82-1.08)	0.375
Age 60-69 years old, females (n = 51,028)						
Statin prescription (1 year)	1.04 (1.00-1.08)	0.079	0.93 (0.89-0.97)	0.001*	0.95 (0.90-0.99)	0.017*
Age 70-79 years old, males (n = 8,778)						
Statin prescription (1 year)	0.96 (0.89-1.04)	0.355	0.94 (0.86-1.02)	0.138	0.94 (0.86-1.03)	0.184
Age 70-79 years old, females (n = 20,843)						
Statin prescription (1 year)	1.89 (1.75-2.04)	<0.001*	1.42 (1.30-1.55)	<0.001*	1.43 (1.31-1.56)	<0.001*
Age ≥ 80 years old, males (n = 1,682)						
Statin prescription (1 year)	1.10 (0.92-1.31)	0.290	1.08 (0.89-1.32)	0.438	1.09 (0.89-1.33)	0.423
Age ≥ 80 years old, females (n = 2,831)						
Statin prescription (1 year)	1.78 (1.45-2.18)	<0.001*	1.30 (1.04-1.64)	0.024*	1.27 (1.01-1.60)	0.044*

Abbreviations: CCI, Charlson comorbidity index; DBP, diastolic blood pressure; SBP, systolic blood pressure

* Logistic regression, Significance at $P < 0.05$

† A model 1 was adjusted for age, sex, income, and region of residence

‡ A model 2 was adjusted for model 1 plus dyslipidemia history, total cholesterol, SBP, DBP, and fasting blood glucose

§ A model 3 was adjusted for model 2 plus obesity, smoking, alcohol consumption, and CCI scores.

Table S2. Odds ratios (95% confidence interval) of the date of hydrophilic statin prescription (1 year) for osteoporosis with subgroup analyses according to age, sex, income, and region of residence

Characteristics	Odds ratios for osteoporosis					
	Model 1†	P-value	Model 2‡	P-value	Model 3§	P-value
Total participants (n = 137,184)						
Statin prescription (1 year)	1.01 (0.95-1.08)	0.720	0.94 (0.88-1.00)	0.042*	0.95 (0.89-1.01)	0.087
Age < 60 years old, males (n = 4,178)						
Statin prescription (1 year)	1.09 (0.76-1.58)	0.635	0.91 (0.62-1.33)	0.621	0.92 (0.63-1.35)	0.683
Age < 60 years old, females (n = 74,763)						
Statin prescription (1 year)	0.87 (0.79-0.96)	0.007*	0.86 (0.77-0.95)	0.003*	0.86 (0.78-0.96)	0.005*
Age ≥ 60 years old, males (n = 13,516)						
Statin prescription (1 year)	1.01 (0.87-1.17)	0.929	0.97 (0.84-1.13)	0.731	0.98 (0.85-1.14)	0.807
Age ≥ 60 years old, females (n = 44,727)						
Statin prescription (1 year)	1.46 (1.29-1.65)	<0.001*	1.10 (0.97-1.24)	0.145	1.12 (0.99-1.27)	0.070
Low income (n = 66,340)						
Statin prescription (1 year)	1.04 (0.94-1.14)	0.486	0.95 (0.86-1.05)	0.316	0.96 (0.87-1.06)	0.431
High income (n = 70,844)						
Statin prescription (1 year)	0.99 (0.91-1.07)	0.809	0.92 (0.85-1.00)	0.059	0.93 (0.86-1.01)	0.103
Urban (n = 54,018)						
Statin prescription (1 year)	1.00 (0.91-1.09)	0.913	0.91 (0.83-1.00)	0.047*	0.92 (0.84-1.01)	0.065
Rural (n = 83,166)						
Statin prescription (1 year)	1.04 (0.96-1.13)	0.372	0.97 (0.89-1.05)	0.429	0.98 (0.90-1.07)	0.598

Abbreviations: CCI, Charlson comorbidity index; DBP, diastolic blood pressure; SBP, systolic blood pressure

* Logistic regression, Significance at P < 0.05

† A model 1 was adjusted for age, sex, income, and region of residence

‡ A model 2 was adjusted for model 1 plus dyslipidemia history, total cholesterol, SBP, DBP, and fasting blood glucose

§ A model 3 was adjusted for model 2 plus obesity, smoking, alcohol consumption, and CCI scores.

Table S3. Odds ratios (95% confidence interval) of the date of lipophilic statin prescription (1 year) for osteoporosis with subgroup analyses according to age, sex, income, and region of residence

Characteristics	Odds ratios for osteoporosis					
	Model 1†	P-value	Model 2‡	P-value	Model 3§	P-value
Total participants (n = 137,184)						
Statin prescription (1 year)	1.05 (1.02-1.08)	0.001*	0.96 (0.93-0.99)	0.016*	0.98 (0.95-1.01)	0.204
Age < 60 years old, males (n = 4,178)						
Statin prescription (1 year)	1.11 (0.95-1.31)	0.199	0.99 (0.83-1.18)	0.888	1.01 (0.85-1.22)	0.882
Age < 60 years old, females (n = 74,763)						
Statin prescription (1 year)	0.84 (0.81-0.88)	<0.001*	0.80 (0.76-0.84)	<0.001*	0.81 (0.77-0.85)	<0.001*
Age ≥ 60 years old, males (n = 13,516)						
Statin prescription (1 year)	0.98 (0.91-1.06)	0.616	0.95 (0.88-1.03)	0.186	0.96 (0.88-1.04)	0.298
Age ≥ 60 years old, females (n = 44,727)						
Statin prescription (1 year)	1.85 (1.74-1.96)	<0.001*	1.40 (1.32-1.49)	<0.001*	1.41 (1.33-1.50)	<0.001*
Low income (n = 66,340)						
Statin prescription (1 year)	1.05 (1.01-1.10)	0.013*	0.95 (0.91-1.00)	0.043*	0.97 (0.93-1.02)	0.260
High income (n = 70,844)						
Statin prescription (1 year)	1.04 (1.01-1.08)	0.021*	0.97 (0.93-1.01)	0.137	0.98 (0.94-1.03)	0.444
Urban (n = 54,018)						
Statin prescription (1 year)	1.02 (0.98-1.07)	0.265	0.93 (0.88-0.97)	0.001*	0.94 (0.90-0.99)	0.009*
Rural (n = 83,166)						
Statin prescription (1 year)	1.08 (1.04-1.12)	<0.001*	1.00 (0.96-1.04)	0.981	1.02 (0.98-1.06)	0.434

Abbreviations: CCI, Charlson comorbidity index; DBP, diastolic blood pressure; SBP, systolic blood pressure

* Logistic regression, Significance at P < 0.05

† A model 1 was adjusted for age, sex, income, and region of residence

‡ A model 2 was adjusted for model 1 plus dyslipidemia history, total cholesterol, SBP, DBP, and fasting blood glucose

§ A model 3 was adjusted for model 2 plus obesity, smoking, alcohol consumption, and CCI scores.
